# Fluorescence and Near-Infrared Light for Detection of Secondary Caries: A Systematic Review

**DOI:** 10.3390/dj11120271

**Published:** 2023-11-28

**Authors:** Dimitrios Spagopoulos, Stavroula Michou, Sotiria Gizani, Eftychia Pappa, Christos Rahiotis

**Affiliations:** 1 Department of Operative Dentistry, National and Kapodistrian University of Athens, 11527 Athens, Greece; dimspag@dent.uoa.gr (D.S.); effiepappa@dent.uoa.gr (E.P.); 2 Department of Odontology, University of Copenhagen, DK-2200 Copenhagen, Denmark; stavroula.michou@sund.ku.dk; 3 Department of Pediatric Dentistry, National and Kapodistrian University of Athens, 11527 Athens, Greece

**Keywords:** secondary caries, dental restorations, fluorescence, near-infrared imaging, diagnostic accuracy, and systematic review

## Abstract

Background: Early detection of secondary caries near dental restorations is essential to prevent further complications. This systematic review seeks to evaluate the sensitivity of fluorescence and near-infrared (NIR) imaging techniques for detecting secondary caries and to provide insight into their clinical utility. Methods: A comprehensive search strategy was used to select studies from seven databases, emphasizing diagnostic accuracy studies of secondary caries detection using fluorescence and NIR imaging techniques. The Quality Assessment of Diagnostic Accuracy Studies-2 (QUADAS-2) instrument assessed bias risk and practicality. Two evaluators performed data extraction, screening, and quality assessment independently. Results: From 3110 initial recordings, nine studies were selected for full-text analysis. Wide variations in sensitivity (SE) and specificity (SP) values were reported across the studies. These studies exhibited variable SE and SP values, and the findings highlighted the importance of method selection based on clinical context. This systematic review underlines the potential for fluorescence and NIR imaging to detect secondary caries. However, results from different studies vary, indicating the need to consider additional variables such as restoration materials. Conclusions: Although these technologies exhibit potential for detecting caries, our research underscores the complex procedure of identifying secondary caries lesions. It is a continuous necessity for progress in dental diagnostics to promptly identify secondary caries lesions, particularly those in proximity to tooth-colored ones.

## 1. Introduction

Dental caries result from interactions between tooth structure, biofilm accumulation, carbohydrates, sugars, salivary, and host factors [1]. Whenever the demineralization exceeds the remineralization in some regions of the tooth’s surface, it can lead to the development of initial caries. However, the carious disease process can be controlled and sometimes arrested or healed if diagnosed and treated early. Following the same principle, caries lesions can be created adjacent to the restoration’s margins [2]. Many authors have proposed that secondary caries, or caries associated with restorations and sealants (CARS), are linked to gaps between the restoration and the tooth surface [3]. In contrast, Kidd et al. showed that secondary caries refer to initial caries adjacent to restorations [4]. Secondary caries or lesions adjacent to restorations are the major late complications of dental restorations. [2] Secondary caries must be identified early to apply preventive protocols. Usually, patients with caries adjacent to restorations are retreated with more extensive restorations at the expense of the dental structure, which can even lead to tooth extraction [5]. In addition, replacing these restorations significantly impacts the expenditure on the patient’s health care, as they seem to account for a considerable amount of the everyday clinical work [6]. The critical component in secondary caries detection/diagnosis is detecting it early to apply preventive and minimally invasive strategies. The visual-tactile and radiographic examinations are the most commonly used methods for secondary caries assessment [6]. However, visual-tactile examination is challenging for clinicians because the lesions can be misinterpreted with minor defects such as discolored marginal staining and residual carious lesions left behind when applying minimally invasive techniques [7]. On the other hand, although the digital radiographic method emits little radiation, it leads to misinterpretations due to the difficulty in differentiating between the restorative material and its interface with tooth structure [8]. To overcome these difficulties, newer approaches based on the optical detection of caries have also been suggested [9]. These approaches are used to examine changes in optical characteristics and light scattering in hard tooth tissues in the presence of caries [10]. However, even though various optical caries detection technologies have promised diagnostic performance, they all have drawbacks. Furthermore, many optical caries detection systems are designed for research purposes and could be more user-friendly for clinical usage [10,11].

Optical caries detection methods include those based on fluorescence and near-infrared (NIR) transillumination or reflectance (back-scatter imaging).

Fluorescence-based optical caries detection assesses the autofluorescence emitted by the hard dental tissues when excited with blue-violet light. [9,12]. Wavelength (λ) at 370 nm was initially employed. However, a longer wavelength at 405–450 nm was adopted later by different devices. When this blue-violet light excites the sound, dental tissues emit bright green fluorescence, while demineralized tissue absorbs/scatters more light and emits reduced green fluorescence.

Furthermore, orange-red autofluorescence is emitted by bacterial metabolites. High accuracy rates have been presented with this technology, but mostly on non-proximal surfaces [12,13,14]. Nevertheless, because it presents similar accuracy to a radiographic evaluation in diagnosing caries on proximal surfaces, it can be a potential alternative, especially when there is a contraindication for ionizing radiation [15]. However, adjacent to amalgam restoration, scattering and reflection can alter the accuracy of such devices [16].

NIR light, used at various wavelengths, has emerged as an essential technique for detecting dental cavities in modern dentistry. This ground-breaking system uses NIR light for transillumination and back-scatter imaging (reflection). Transillumination transports NIR light through the teeth, with dental caries lesions affecting the light’s scattering and absorption characteristics, resulting in shadows or dark patches in the image. On the other hand, back-scatter imaging puts NIR light onto the tooth surface and analyzes the reflected light, with caries lesions influencing the scattering properties and forming different patterns. These methods have several advantages, including early detection, non-invasiveness, immediate results, and no radiation exposure [12].

Even though this technology has been well established and studied for initial caries detection, either occlusal or interproximal, heterogeneity, unconfirmed data, and subjectivity according to detective tools in the current published clinical research all fuel future initiatives to assess the existing evidence in the field comprehensively.

This systematic review aims to compare all in vivo and in vitro studies to evaluate the sensitivity (SE) and specificity (SP) of fluorescence and/or NIR imaging methods for detecting and assessing secondary caries.

## 2. Materials and Methods

This systematic review answered the question, “What is the accuracy of fluorescence and NIR imaging methods as tools for secondary caries detection, regardless of the type of teeth, dentition, and age of the patient population?”.

The protocol of the Cochrane Collaboration was followed in this review with the Review Manager (RevMan v5.3, the Cochrane Collaboration, Copenhagen, Denmark) [17]. The reporting scheme of the review was aligned with the recommendations of the latest PRISMA statement for diagnostic test accuracy studies [18] and the PICO framework [19]. The present systematic review was registered on the PROSPERO platform (DOI number CRD42023412721).

### 2.1. Search Strategy

The selection process for studies included in the systematic review started with a comprehensive search of seven databases: PubMed, Scopus, Cochrane, Google Scholar and Grey Literature, Web of Science, and Embrase. The search strategy was developed using relevant keywords and search terms: (secondary caries OR caries adjacent to restorations OR recurrent caries OR caries related to restorations) AND (detection OR diagnosis) AND (NIR OR transillumination OR infrared OR reflection OR reflectance OR IR OR fluorescence). Filter was used to ensure the research dates from 2000–2023. Two independent reviewers (D.S., S.M.) screened the titles and abstracts of the retrieved articles to assess their eligibility for inclusion in the review. Full-text articles of potentially eligible studies were retrieved and assessed for eligibility based on pre-defined inclusion and exclusion criteria. Any discrepancies between the two reviewers were resolved through discussion, with a third reviewer (C.R.) consulted if necessary.

This systematic review of the literature included in vitro and in vivo diagnostic studies that tested the diagnostic accuracy and/or reliability of detecting and assessing secondary caries in human teeth. In vivo studies were included regardless of the age of the population or the number of included patients or teeth. The following index tests were included in the search: visual-tactile examination and bitewings, fluorescence, and/or NIR imaging methods. The included in vitro studies had to use any well-established histological technique (e.g., slices, grinding, hemisection, or microradiography) to validate the ‘true’ caries extension; otherwise, the studies were excluded. The inclusion criteria were:

- Studies that evaluate the diagnostic accuracy of fluorescence and/or NIR imaging methods for detecting and assessing secondary caries in human teeth
- Studies that compare fluorescence and/or NIR imaging methods with conventional diagnostic methods such as visual-tactile examination and radiography
- Studies that report sensitivity (SE), specificity (SP), positive predictive value, negative predictive value, or area under the receiver operating characteristic curve (AUC) for fluorescence and/or NIR imaging methods
- Studies that are published in the English language
- The exclusion criteria applied during the article selection process were as follows:
- Studies not written in the English language were excluded from the review
- Studies that did not evaluate the diagnostic accuracy of fluorescence and NIR imaging methods for detecting and assessing secondary caries in human teeth were excluded
- Studies lacking essential diagnostic accuracy measures, such as sensitivity (SE), specificity (SP), positive predictive value, negative predictive value, or area under the receiver operating characteristic curve (AUC) for fluorescence and/or NIR imaging methods, were excluded

### 2.2. Data Extraction

Data were extracted from the included studies using a standardized data extraction form. The extracted data included study characteristics, imaging methods, comparator methods, and diagnostic accuracy measures. Patient-reported outcome and cost-effectiveness data were also extracted where available. The data extraction process was independently performed by two reviewers, with any discrepancies resolved through discussion.

All extracted data were entered into a database and checked for accuracy and completeness. The process is documented and recorded to ensure the transparency and reproducibility of the review. This rigorous approach to study selection and data extraction helped ensure that the systematic review is comprehensive, objective, and of high quality, increasing its findings’ reliability and validity.

### 2.3. Screening and Eligibility Check

Two reviewers (D.S., S.M.) screened the titles and abstracts independently. The reviewers were not blinded to the authors’ names, institutions, journals, or publication results. All records were cross-checked concerning the initially consented inclusion and exclusion criteria. Discussions with an experienced researcher (C.R.) continuously resolve doubts or disagreements.

### 2.4. Risk of Bias Assessment (RoB)

In the systematic review, the studies were assessed for quality using the Quality Assessment of Diagnostic Accuracy Studies (QUADAS-2) tool, designed to evaluate the risk of bias and applicability of diagnostic accuracy studies. The tool assesses four domains: patient selection, index test, reference standard, and flow and timing. Each domain was evaluated for concerns regarding applicability and risk of bias.

Two independent reviewers (D.S., S.M.) used the QUADAS-2 tool to assess the risk of bias and applicability of the included studies. In cases of discrepancies between the reviewers, they discussed resolving them. This approach enhanced the reliability and validity of the systematic review’s findings. The studies were investigated in subgroups based on the type of diagnostic method used for the detection and assessment of secondary caries. Specifically, the studies that use fluorescence imaging were compared to those that use NIR imaging. A subgroup analysis was prepared to compare the diagnostic accuracy of fluorescence and NIR imaging. The studies were divided into two subgroups based on the imaging technique used, and the diagnostic accuracy of each subgroup will be compared.

By investigating subgroups and covariates, the aim was to identify potential sources of heterogeneity and explore factors that may affect the diagnostic accuracy of fluorescence and NIR imaging. This would provide a more comprehensive understanding of the diagnostic methods for detecting and assessing secondary caries, which can be used to guide clinical practice and future research in this field.

## 3. Results

**Figure 1:**
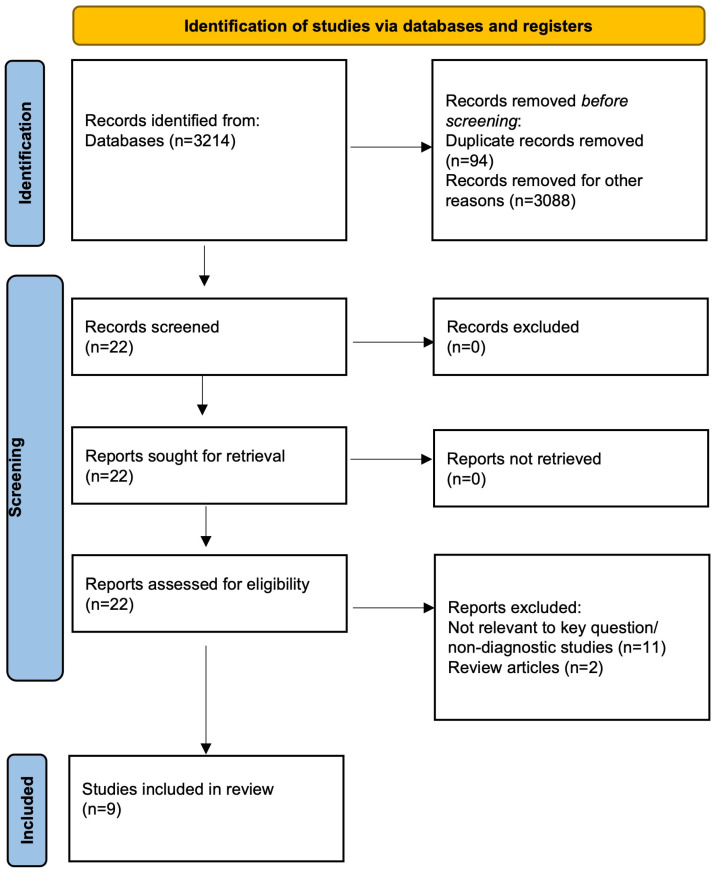
The Prisma flow chart of the study.

Out of 3214 unique records filtered using an electronic database, 94 were duplicates from the databases, and 3088 were irrelevant to the subject of the systematic review. Twenty-two articles were selected for further screening based on their titles and abstracts following the identification phase. The screening criteria included relevance to the study topic and diagnostic accuracy studies of secondary caries detection using fluorescence and NIR imaging techniques. The eligibility phase involved a full-text analysis of the 22 selected articles to assess their suitability based on pre-defined inclusion and exclusion criteria. Articles meeting the eligibility criteria were included in the systematic review. Nine studies were left for a full-text evaluation by the qualifying criteria (Figure 1).

One study was in vivo [16], and the rest were in vitro. In two studies, the restoration material was resin composite [20,21]; in one study, there was resin-modified glass ionomer cement; and in five studies, the restorative material was amalgam [22]. Table 1 showed the results of SE and SP of the studies.

We observed a range of sensitivity (SE) and specificity (SP) values for various dental caries detection methods in our systematic review. Ando et al. reported SE values between 18 and 91% and SP values between 21 and 96%, recommending quantitative light-induced fluorescence (QLF), DIAGNOdent, and visual inspection over gap inspection [23]. Bamzahim et al. discovered SE values of 60% for DIAGNOdent, 56% for X-ray, and 44% for visual inspection, with corresponding SP values of 82%, 92%, and 96% [16]. Rodrigues et al. reported SE values ranging from 23% to 89% and variable SP values, with the LF pen providing comparable accuracy to conventional techniques but with greater examiner reproducibility [21]. Neuhaus et al. found SE values between 9 and 81% for bitewing (BW) and 41 and 81% for LF, as well as SP values between 95 and 98% for BW and 60 and 80% for LF. In addition, they discovered that LF excelled in crown evaluation, while BW performed comparably in root evaluation [24]. Lenzi et al. reported SE values between 62–78%, 42–71%, and 71–78% for QLF, X-ray, and visual inspection, respectively, with SP values between 63–82% for QLF, 54–82% for X-ray, and 64–90% for visual inspection [25]. Diniz et al. demonstrated that SP and SE results varied across methods [20]. Abrahams et al. reported SE values between 45–94% and 95–100% for Canary System and DIAGNOdent, and SP values between 54–100% and 85–100% for Canary System and DIAGNOdent, respectively [26]. In another study, SE values ranged from 18 to 69% for ICDAS, SPECTRA, and DIAGNOdent, and from 90 to 100% for Canary System. SP values ranged from 52 to 66% for ICDAS, 61% for spectra, 66 to 92% for DIAGNOdent, and 71 to 92% for Canary System [22].

The Cohen’s K coefficient for inter-rater reliability of Rob analysis between the two evaluators was near perfect agreement (kappa = 0.91). In the discrepancies, they discussed and agreed.

Table 2 shows the findings of the visual analysis of in vitro validation experiments on occlusal surfaces, emphasizing various areas of risk of bias. All the studies, except one [26], used fluorescence devices to diagnose secondary caries. Two independent reviewers utilized the QUADAS-2 technique to assess the risk of bias and the applicability of the included research. The tool includes signaling questions that address several types of bias in the design and execution of studies.

In terms of potential selection bias, most of the papers assessed were ambiguous (−). This implies that the studies did not sufficiently describe how patients were chosen for inclusion in detail. Similarly, selecting individual teeth for inspection was primarily opaque (−), showing a lack of transparency in the process. Most investigations were negative (−) regarding the caries spectrum, meaning that the caries spectrum was not sufficiently accounted for or reported in the validation studies. This has the potential to add bias to the results. Furthermore, the sample size was often small (−), indicating that the statistical power of the research was limited.

The index test bias differed between investigations. While some studies received good (+) ratings, indicating a low risk of bias in judging index test accuracy, others received negative (−) ratings, indicating a higher risk of bias. Similarly, the bias in the reference tests used to evaluate the index test was predominantly negative (−), indicating a potential danger of bias in the reference tests used to evaluate the index test.

Most of the verification bias (differences in the application of the reference standard) was negative (−), indicating a high risk of bias in this aspect. A few studies, however, were evaluated as questionable (?), indicating the need for greater clarity.

Outcome bias, or differences in how outcomes are determined or interpreted, was generally negative (−), indicating a likelihood of bias. A few studies, however, were scored as positive (+), indicating minimal bias in the reporting or interpretation of results.

Other sources of bias, such as blinding bias, calibration bias, incorporation bias, partial verification bias, differential verification bias, bias in the analysis, validity bias, and reproducibility bias, varied across studies, with ratings ranging from positive (+) to negative (−) and uncertain (?).

**Figure 2:**
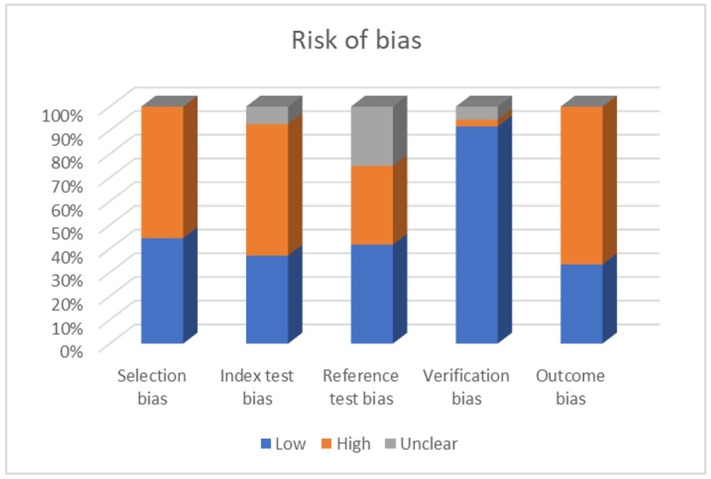
The percentage of RoB per category.

The percentage of RoB is shown in Figure 2.

When bias was assessed across the examined studies, it was discovered that around 44% had a low risk of selection bias, indicating trust in participant or sample selection, whereas 56% had a high risk. Regarding index test bias, around 40% were classified as low risk, while 60% were classified as high risk. Regarding reference test bias, around 42% had a low risk, 33% had a high risk, and 25% had an unknown status. Verification bias was generally modest, with around 89% of studies showing low risk, 3% showing high risk, and 5% remaining ambiguous. These percentages provide useful information on the distribution of bias risks across studies, highlighting areas where methodological improvements may be required to increase research validity.

## 4. Discussion

This systematic review reported a high risk of bias in the existing literature. Considering the extensive analysis in the introduction, assessing secondary caries lesions utilizing fluorescence or NIR equipment poses a subtle issue in modern dentistry practice. The lack of studies addressing this diagnostic component highlights a crucial gap in the available knowledge. While the research included in this review investigated various approaches and technologies, the findings highlight both opportunities and limitations in their application for identifying secondary caries lesions close to dental restorations.

Numerous diagnostic techniques, such as quantitative light-induced fluorescence (QLF), DIAGNOdent, ocular assessment, radiographic examination, laser fluorescence (LF) pen, and others, were used in the papers that made up this study. Each study sought to compare the effectiveness of these techniques to a reference standard, frequently assessing diagnostic accuracy using statistical approaches, including receiver operating characteristic (ROC) curves.

Interestingly, these devices show promising results and should be considered when re-examination and monitoring of lesions occur without exposing patients to ionizing radiation. However, it is essential to note that their diagnostic accuracy may be modified by factors such as the restoration material employed, with potential limitations in detecting lesions next to amalgam restorations. The significance of addressing potential limits in identifying secondary caries near amalgam restorations is emphasized by previous research, which supports the recommendation to refinish and polish the proximal areas of these restorations before examination with fluorescence devices; otherwise, these will result in false positive results [24]. The presence of accumulated stain or bacteria has the potential to impact devices that work with fluorescence, underscoring the significance of selecting appropriate diagnostic tools, particularly in the context of various restoration materials. This discovery emphasizes the importance of dentists carefully weighing the selection of diagnostic instruments depending on the individual clinical scenario.

The systematic review’s findings and trends addressed a wide range of topics. The SE and SP reported a wide range. While some studies showed encouraging results, such as high SE and SP, others raised questions regarding the restrictions and usability of detective techniques, especially in specific clinical circumstances or when spotting secondary caries adjacent to dental restorations. Factors like the restorative materials, the condition of dental surfaces, and the duration of sample storage also influenced the precision of these diagnostic techniques. Ando et al. [23] studied various diagnostic tools for detecting secondary caries and found SE and SP values ranging from 18% to 91% and 21% to 96%, respectively. The results of their study indicate that the use of visual examination in conjunction with diagnostic instruments such as DIAGNOdent may exceed the gap inspection by clinical examination. Bamzahim et al. [16] conducted a study to evaluate the diagnostic performance of DIAGNOdent, X-ray, and visual inspection methods in detecting recurrent caries. The authors reported SE and SP values for each method, with DIAGNOdent having an SE of 60% and SP of 82%, X-ray having an SE of 56% and SP of 92%, and visual inspection having an SE of 44% and SP of 96%. These findings highlight the varying effectiveness of these methods in diagnosing recurrent caries. Rodrigues et al. [21] presented findings that demonstrated a broad spectrum of SE (ranging from 23% to 89%) and SP (ranging from 0% to 100%). Notably, the utilization of a light fluorescence pen (Lf pen) yielded comparable accuracy to traditional techniques while also improving the reproducibility of results by examiners. The study conducted by Neuhaus et al. presented data on the SE and SP values for different types of lesions, suggesting that LF and crown diagnoses may have advantages over BW and root caries diagnostics [24]. The study conducted by Lenzi et al. investigated the efficacy of quantitative light-induced fluorescence (QLF), X-rays, and visual examination as diagnostic methods [25]. The results demonstrated that each approach exhibited different ranges of SE and SP.

Furthermore, Simon et al. [27] highlighted the potential efficacy of near-infrared (NIR) imaging as a screening modality. In their study, Diniz et al. analyzed the SE and SP values associated with several diagnostic methods, such as ICDAS, LF, QLF, and MID [20]. The authors specifically highlighted the observed discrepancies in diagnostic effectiveness among these approaches. In conclusion, Abrahams et al. [26] presented the SE and SP values for Canary, DIAGNOdent, and ICDAS, underscoring the varied diagnostic results observed in cases of recurrent caries. These results highlight the significance of carefully choosing suitable diagnostic methods and techniques that are customized to specific clinical scenarios to diagnose recurrent caries effectively and precisely.

To the best of our knowledge, this is, to date, the first comprehensive systematic review assessing the fluorescence and NIR infrared detection methods for secondary caries. The protocol of this review was a priori registered and followed a pre-defined methodology that safeguarded against selective reporting and considerable deviations from the original design. On the other hand, some limitations do exist. More than 41% of the included studies had an unclear or high risk of bias regarding the reference standard, and more than 66% showed a high risk of bias regarding the outcome. The fact that none of the studies could be included in the meta-analysis due to the heterogeneity of the data is also a considerable limitation of this study. Most of the studies in this systematic review mainly focused on examining amalgam restorations, with only one study investigating resin-modified glass ionomer restorations [26]. This phenomenon may elucidate the variability observed in the SE and SP values across the research examined in this study. Only two studies examined extracted teeth in conjunction with direct composites, even though it is the material of choice for direct restorations. Additionally, there is an extensive selection of composite materials on the market with varying fluorescence levels. The lack of information regarding the composite restoration employed in the extracted tooth studies affects the ability to obtain conclusive results on the accuracy of fluorescent devices in detecting recurrent caries. As for any diagnostic imaging technology, the interpretation of grayscale images by the NIR method is limited by interrater disagreement. The use of magnification can help with correct detection. However, none of the above-mentioned studies compare magnification as a factor in visual inspection.

Overall, this discussion provides insight into the complications surrounding the identification of secondary caries lesions and emphasizes the need for additional research and developments in dental diagnostics.

## 5. Conclusions

In summary, this systematic review highlights the potential for near-infrared (NIR) and fluorescence devices in detecting secondary caries. It emphasizes the criticality of conducting thorough evaluations of study design, material conditions, and diagnostic criteria. Although these technologies exhibit potential in detecting caries, our research underscores the complex procedure of identifying secondary caries lesions. Further studies should give priority to the resolution of these obstacles to improve current diagnostic techniques and examine innovative methodologies. The optimization of diagnostic instruments for distinct clinical situations, with particular attention to the plethora of restoration materials, is of critical significance in the field of dentistry to achieve precise and predictable outcomes. This conclusion is in greater accordance with the principal objective of the research, underscoring the continuous necessity for progress in dental diagnostics to promptly identify secondary caries lesions, particularly those in proximity to tooth-colored ones.

## Figures and Tables

**Table 1:** Summarizes the results of SE and SP of the studies.

Study	Outcome
Ando et al. [23]	SE 18–91% SP 21–96% SE: qlf, diagnodent similar to visual inspection better then gap inspection
Bamzahim et al. [16]	SE diagnodent 60%, xray 56%, visual 44% SP diagnodent 82%, xray 92%, visual 96%
Rodrigues et al. [21]	SE 23–89% SP 0–100% Lf pen similar accuracy to conventional but higher examiner reproducibility
Neuhaus et al. [24]	SE BW 9–30% LF 41–81% SP BW 95–98% LF 60–80% Crown LF better than BW, Root LF similar to BW
Lenzi et al. [25]	SE QLF 62–78% Xray 42–71% visual 71–78% SP QLF 63–82% Xray 54–82% visual 64–90%
Diniz et al. [20]	SP ICDAS 70–72% LF 83–84% QLF 29–53% MID 87–92% SE ICDAS 20–71% LF 5–70% QLF 61–84% MID 5–54%
Abrahams et al. [26]	SE canary 95–100% diagnodent 45–94% SP canary 85–100% diagnodent 54–100%
Abrahams et al. [22]	SΕ ICDAS 34% spectra 34% diagnodent 18–69% canary 90–100% SP ICDAS 52% spectra 61% diagnodent 66–92% canary 71–92%

**Table 2:** The RoB analysis of the studies.

Visual Examination of In Vitro Validation Studies on Occlusal Surfaces	Signaling Questions															
	Selection Bias				Index Test Bias			Reference Test Bias				Verification Bias			Outcome Bias	
	Patient Selection	Teeth Selection	Caries Spectrum	Sample Size	Test Criteria	Blinding Bias	Calibration Bias	Test Criteria	Blinding Bias	Calibration Bias	Incorporation Bias	Partial Ver. Bias	Differential Ver. bias	Bias in the Analysis	Validity Bias	Reproducibility Bias
Ando (2004) [23]	x	+	+	−	+	+	−	+	?	−	+	+	+	+	+	−
Bemzahim (2005) [16]	−	+	−	−	+	+	−	+	+	−	+	+	+	+	−	−
Rodrigues (2010) [21]	x	−	+	−	−	+	−	+	+	−	+	+	+	+	−	+
Neuhaus (2012) [24]	x	−	+	−	−	−	−	+	−	−	+ ?	+	+	+	+	+
Lenzi (2016) [25]	x	+	+	−	+	−	−	+	−	−	+	+	+	+	−	+
Simon (2015) [26]	x	−	−	−	−	−	−	−	−	−	+	+	+	−	−	−
Diniz (2016) [20]	x	−	+	−	+	+	−	+	−	−	+	+	+	+	−	+
Abrahams (2017) [26]	x	+	+	−	+	?	−	?	?	?	?	+	+	+	−	−
Abrahams (2018) [22]	x	+	+	−	+	?	−	?	?	?	?	+	+	+	−	−

Legend: + = Low risk of bias (Yes); − = High risk of bias (Probably No, No); ? = Unclear (No information, Incomplete reporting, Probably Yes) x = Question for in vivo study only.
